# Timing of corticosteroid-sparing therapies in non-infectious uveitis: a population-based cohort study from Denmark

**DOI:** 10.1038/s41433-026-04728-2

**Published:** 2026-08-11

**Authors:** Viktor Skalkhøj Oest, Bawan Halgurd, Yousif Subhi, Maria Vittoria Cicinelli, Piergiorgio Neri, Henrik Vorum, Lasse Jørgensen Cehofski, Marie Ørskov

**Affiliations:** 1 Department of Ophthalmology, Aalborg University Hospital, Aalborg, Denmark; 2 Department of Ophthalmology, University Hospital of Southern Denmark, Sønderborg, Denmark; 3 Department of Regional Health Research, University of Southern Denmark, Odense, Denmark; 4 School of Medicine, Vita-Salute, San Raffaele University, Milano, Italy; 5 Department of Ophthalmology, IRCCS San Raffaele Scientific Institute, Milano, Italy; 6 Eye Institute, Cleveland Clinic Abu Dhabi, Abu Dhabi, United Arab Emirates; 7 Cleveland Clinic Lerner College of Medicine, Case Western Reserve University, Cleveland, OH USA; 8 Department of Clinical Medicine, Aalborg University, Aalborg, Denmark

**Keywords:** Uveal diseases, Epidemiology

## Abstract

**Background:**

Non-infectious uveitis (NIU) is a group of potentially vision-threatening diseases where timely management is critical, yet data on the timing and sequencing of therapeutic interventions remain limited. Here, we evaluated real-world patterns and delays in initiating key NIU therapies over time.

**Methods:**

A total of 1218 patients with NIU referred to a hospital-based ophthalmology clinic in the North Denmark Region between 2010 and 2024 were included. The timing of treatment initiation with periocular corticosteroid injections, intravitreal dexamethasone implants (IDI), systemic prednisolone, or disease-modifying antirheumatic drugs (DMARDs), was analysed using cumulative incidence and incidence rates. Associations between patient characteristics and treatment initiation were assessed using Cox proportional-hazards models.

**Results:**

Systemic prednisolone and periocular corticosteroid injections were initiated rapidly, with most treatments administered within the first three months after referral. IDI treatment was initiated later, with few patients receiving IDI during the first year. Initiation of DMARD therapy occurred gradually over the first year. At five years, cumulative incidences were: periocular corticosteroid injections 6.2% (95% CI: 4.9–7.9), IDI 2.6% (95% CI: 1.8–3.8), systemic prednisolone 11.3% (95% CI: 9.6–13.3), and DMARDs 4.1% (95% CI: 3.1–5.5). IDI use was more likely in intermediate, posterior, and panuveitis while DMARDs were more frequently initiated in patients with bilateral disease.

**Conclusion:**

Systemic and periocular corticosteroids injections were used frequently and early, while IDI and DMARDs were introduced later. These findings may reflect the clinical characteristics of this cohort and potential barriers to timely corticosteroid-sparing therapies and highlight the importance of continued efforts to optimise timing of treatment in the management of NIU.

## Introduction

Uveitis comprises a diverse group of potentially sight-threatening inflammatory diseases affecting the uveal tract, as well as adjacent ocular structures. It can occur in both adults and children and is broadly categorised as either infectious or non-infectious [1]. Non-infectious uveitis (NIU) may arise in the context of systemic diseases or as an isolated ocular condition without identifiable systemic association, and a substantial proportion of cases remain idiopathic, with reported proportions ranging from 27%–51% [1,2]. Management of NIU typically involves immunomodulatory therapies, including periocular corticosteroid injections, intravitreal corticosteroid implants, systemic corticosteroids, disease-modifying antirheumatic drugs (DMARDs), and in selected cases biologic agents such as tumour necrosis factor-alpha (TNF-α) inhibitors [3].

Timely diagnosis and intervention are essential in NIU to preserve vision and prevent permanent structural damage [4]. Understanding real-world treatment patterns and the timing of therapeutic interventions can help identify barriers to optimal care and develop strategies to improve outcomes. However, comprehensive data on the longitudinal use of NIU therapies are limited, as large cohorts are challenging to follow over time.

The Danish National Patient Registry, which offers validated, population-based clinical data, provides a unique opportunity to study the management of NIU over extended periods of time. In this study, we leveraged registry data and detailed medical record review to investigate the timing, frequency, and determinants of therapeutic interventions in NIU, with a focus on identifying delays in initiating systemic corticosteroid-sparing treatments.

## Methods

### Study population

Data was retrieved from the patient registry of the North Denmark Region. The study included 1218 patients with NIU, aged 0–100 years, who were assigned a relevant International Classification of Diseases, 10th Revision (ICD-10), uveitis code and were evaluated at the Department of Ophthalmology, Aalborg University Hospital, between January 1, 2010, and January 1, 2024. The North Denmark Region serves a population of approximately 600,000 people, representing around 10% of the total Danish population. Denmark has a universal healthcare system in which all hospital care, including surgical interventions, is provided free of charge to patients, irrespective of socioeconomic status.

Patients with ophthalmic conditions are typically first assessed by ophthalmologists in private practice, and only cases with more severe or refractory manifestations are referred to hospital-based care. Consequently, the study population reflects individuals with more severe disease manifestations requiring tertiary care. Additionally, the first recorded visit corresponds to the patient’s first hospital-based assessment, and prior treatment in private practice could not be captured. The study was approved by the Research Committee of the North Denmark Region (study number: 2024-006888) and conducted in accordance with the Declaration of Helsinki and the General Data Protection Regulation (GDPR). Informed consent was waived due to the retrospective study design and anonymised data.

### Medical records review

For each patient, clinical data was independently extracted by two researchers through systematic review of medical records. All data was anonymised prior to analysis. Uveitis diagnoses were confirmed by uveitis specialists based on clinical findings. Cases were classified as infectious if clinical features and diagnostic tests supported an infectious aetiology. In the absence of such evidence, uveitis was considered non-infectious. If diagnostic uncertainty remained, cases were provisionally classified as non-infectious unless definitive testing confirmed an infectious aetiology. Any remaining ambiguous cases were adjudicated by a third uveitis specialist. Data collected included age, sex, anatomical classification of uveitis (anterior, intermediate, posterior, or panuveitis), presence of systemic diseases, and uveitis-related treatments (including drug type, dosage, and date of administration). As prior uveitis episodes managed in private ophthalmology practices are not captured in the medical records available for this study, such episodes could not be identified. Consequently, patients were included from the time of their first uveitis diagnosis registered at a Danish hospital. Only therapies initiated for the management of uveitis were included in the analysis.

### Statistical analysis

The primary outcome of this study was the timing of initiation for each uveitis related treatment modality, specifically periocular corticosteroid injections, IDI, systemic prednisolone, and DMARDs, over a five-year follow-up period. Follow-up began at the first  hospital-based uveitis diagnosis, with treatment initiation dates recorded thereafter. Patients remained under observation as additional treatments could be initiated during the follow-up. Timing was evaluated using incidence rates and cumulative incidence functions. Cumulative incidence was estimated with the Aalen-Johansen estimator, accounting for death as a competing risk. Incidence rates were expressed as the number of treatment initiations per 100 person-years, with corresponding 95% confidence intervals (CIs). Continuous variables were presented as mean ± SD or median (IQR) based on their distribution. Secondary outcomes included the identification of clinical factors associated with earlier treatment initiation, including age, sex, uveitis laterality (unilateral or bilateral), anatomical subtype, and presence of systemic disease. These associations were evaluated using Cox proportional-hazards models, where unilateral and anterior uveitis served as reference categories. Hazard ratios (HRs) with 95% CIs were reported to quantify the relative likelihood of earlier treatment initiation based on patient characteristics. All statistical analyses were conducted using Stata version 18 (StataCorp LP, College Station, TX, USA).

## Results

A total of 1218 patients with NIU were included in the study, comprising 639 women (53%). The mean age at first visit was 49.5 ± 19.4 years   with a total of 873 patients (72%) between 18 and 65 years of age. At presentation, NIU was unilateral in 858 patients (70%) and bilateral in 360 patients (30%). Anatomically, uveitis was classified as anterior in 1060 patients (87%), intermediate in 51 patients (4%), posterior in 79 patients (6%), and panuveitis in 28 patients (3%).

Of the 1218 included patients, 203 (16.7%) had a documented systemic disease. Sarcoidosis was the predominant disease in intermediate, posterior, and panuveitis, present in 9.8%, 7.6%, and 14.3%, respectively, compared to 3.1% in anterior uveitis. HLA-B27-associated conditions, including ankylosing spondylitis, inflammatory bowel disease, and juvenile idiopathic arthritis, and the presence of HLA-B27 were predominantly found in anterior uveitis. Most patients across all subtypes had no identifiable systemic disease, reflecting the predominantly idiopathic nature of non-infectious uveitis in this real-world cohort.

Regarding clinical findings at presentation, median BCVA was 0.10 logMAR (IQR 0.00–0.30) among patients with measurable vision, while 91 patients (7.5%) had a vision recorded as finger counting or worse. Keratic precipitates were present in 370 patients (30.4%), posterior synechiae in 217 patients (17.8%), and elevated intraocular pressure (>21 mmHg) in 162 patients (13.3%). During follow-up, 148 patients (12.2%) underwent cataract surgery, and 33 patients (2.7%) underwent glaucoma surgery, in addition to the 191 patients (15.7%) and 22 patients (1.8%) who had prior cataract and glaucoma surgery, respectively, at presentation (Table 1).

**Table 1:** Baseline characteristics and clinical findings at presentation in the non-infectious uveitis population.

Baseline characteristics	Total (*n* = 1218 patients)
Age (years), mean ± SD	49.6 ( ± 19.4)
**Sex**	
Male	579 (47.5%)
Female	639 (52.5%)
**Laterality**	
Unilateral	858 (70.4%)
Bilateral	360 (29.6%)
**Subtype**	
Anterior	1060 (87.0%)
Intermediate	51 (4.2%)
Posterior	79 (6.5%)
Panuveitis	28 (2.3%)
**HLA-B27 status**	
Positive	179 (14.7%)
**Systemic disease**	
Yes	203 (16.7%)
No	1015 (83.3%)
**Type of systemic disease**	
Ankylosing Spondylitis	62 (5.1%)
Sarcoidosis	48 (3.9%)
Juvenile Idiopathic Arthritis	26 (2.2%)
Inflammatory Bowel Disease	23 (1.9%)
Rheumatoid Arthritis	13 (1.1%)
Psoriatic Arthritis	6 (0.5%)
Other	25 (2.0%)
**Clinical findings**	
BCVA, median logMAR	0.10 (IQR 0.00–0.30)
Keratic precipitates	370 (30.4%)
Posterior synechiae	217 (17.8%)
IOP > 21 mmHg	162 (13.3%)
**Prior ocular history**	
Cataract surgery	191 (15.7%)
Glaucoma surgery	22 (1.8%)

BCVA is reported among patients with measurable vision. All clinical findings reflect presence in at least one eye. Systemic disease diagnoses may include findings during follow-up and do not necessarily imply a direct aetiological association with non-infectious uveitis.

*BCVA* best corrected visual acuity, *IOP* intraocular pressure.

**Fig. 1:**
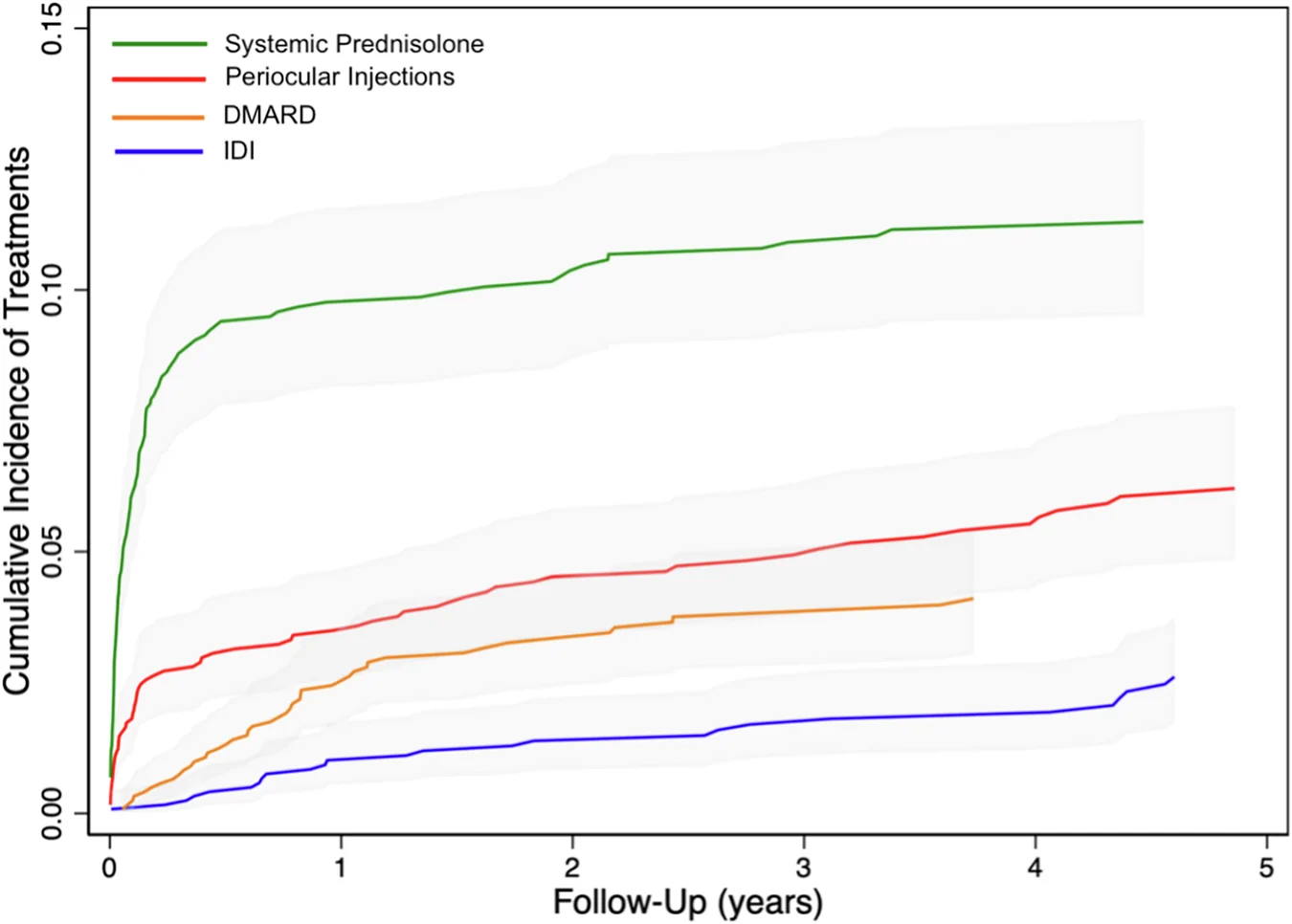
Cumulative incidences of treatment initiation in the non-infectious uveitis population over a 5-year follow-up period with confidence interval (CI): 95%.

During the five-year follow-up period, 66 patients received periocular corticosteroid injections, 26 patients received IDI, 127 were treated with systemic prednisolone, and 45 received DMARD therapy. The initiation of periocular corticosteroid injections and systemic prednisolone occurred early, with a rapid increase in cumulative incidence observed within the first three months. In contrast, the cumulative incidence of IDI increased slowly during the first year, while DMARD use rose steadily over the same period (Fig. 1).

The incidence rate for periocular corticosteroid injections was 1.5 per 100 person-years (95% CI: 1.2–1.9), corresponding to a cumulative incidence of 6.2% (95% CI: 4.9–7.9). For IDI, the incidence rate was 0.5 per 100 person-years (95% CI: 0.4–0.8), with a cumulative incidence of 2.6% (95% CI: 1.8–3.8). Systemic prednisolone had an incidence rate of 3.1 per 100 person-years (95% CI: 2.6–3.7), yielding a cumulative incidence of 11.3% (95% CI: 9.6–13.3) and with a median treatment duration of 3.7 months (IQR 1.5–9.7 months) and a mean of 9.1 months. The incidence rate for DMARD therapy was 1.0 per 100 person-years (95% CI: 0.7–1.3), with a cumulative incidence of 4.1% (95% CI: 3.1–5.5) and a median treatment duration of 17.5 months (IQR 6.5–41.9 months) and a mean of 25.5 months (Fig. 1; Table 2).

**Table 2:** Incidence rates, cumulative incidence, and hazard ratios (HR) for treatment initiation in non-infectious uveitis, stratified by clinical characteristics.

Strata	Events	Person time	Incidence rate (95% CI)	Cumulative incidence (95% CI)	HR (95% CI)
**Periocular injections**	66	4511.7	1.5 (1.2–1.9)	6.2 (4.9–7.9)	
*Laterality*					
Unilateral	37	3114.1	1.2 (0.9–1.6)	4.8 (3.5–6.6)	Reference
Bilateral	29	1397.6	2.1 (1.4–3.0)	9.2 (6.4–12.9)	1.8 (1.1–2.9)
*Subtype*					
Anterior	44	3960.0	1.1 (0.8–1.5)	4.7 (3.5–6.3)	Reference
Other^a^	22	551.7	4.0 (2.6–6.1)	15.9 (10.7–23.4)	3.5 (2.1–5.8)
*Systemic Disease*					
Yes	16	665.7	2.4 (1.5–3.9)	9.7 (6.0–15.5)	0.6 (0.3–1.0)
No	50	3846.0	1.3 (1.0–1.7)	5.6 (4.2–7.3)	Reference
**IDI**	26	4711.1	0.5 (0.4–0.8)	2.6 (1.8–3.8)	
*Laterality*					
Unilateral	11	3265.8	0.3 (0.2–0.6)	1.6 (0.9–2.9)	Reference
Bilateral	15	1445.3	1.0 (0.6–1.7)	5.0 (3.0–8.2)	3.1 (1.4–6.8)
*Subtype*					
Anterior	12	4115.0	0.3 (0.2–0.5)	1.5 (0.9–2.7)	Reference
Other^a^	14	596.1	2.3 (1.4–4.0)	10.0 (6.1–16.5)	8.0 (3.7–17.4)
*Systemic Disease*					
Yes	7	726.3	1.0 (0.5–2.0)	5.1 (2.4–10.5)	0.5 (0.2–1.2)
No	19	3984.7	0.5 (0.3–0.7)	2.2 (1.4–3.5)	Reference
**Systemic Prednisolone**	127	4078.1	3.1 (2.6–3.7)	11.3 (9.6–13.3)	
*Laterality*					
Unilateral	42	3033.5	1.4 (1.0–1.9)	5.3 (3.9–7.1)	Reference
Bilateral	85	1044.6	6.9 (5.4–8.7)	26.2 (21.8–31.4)	5.6 (3.9–8.2)
*Subtype*					
Anterior	62	3780.4	1.6 (1.3–2.1)	6.4 (5.0–8.2)	Reference
Other^a^	65	297.7	21.8 (17.1–27.8)	48 (40.3–57.2)	10.7 (7.6–15.2)
*Systemic Disease*					
Yes	29	583.0	5.0 (3.5–7.2)	16.4 (11.6–23.0)	0.6 (0.4–0.9)
No	98	3495.0	2.8 (2.3–3.4)	10.3 (8.6–12.5)	Reference
**DMARD**	45	4607.9	1.0 (0.7–1.3)	4.1 (3.1–5.5)	
*Laterality*					
Unilateral	8	3254.2	0.2 (0.1–0.5)	1.1 (0.6–2.2)	Reference
Bilateral	37	1353.7	2.7 (2.0–3.8)	10.8 (8.0–14.7)	11.2 (5.2–23.9)
*Subtype*					
Anterior	20	4070.8	0.5 (0.3–0.8)	2.1 (1.4–3.3)	Reference
Other^a^	25	537.0	4.7 (3.1–6.9)	16.5 (11.5–23.5)	8.9 (4.9–16.0)
*Systemic Disease*					
Yes	9	708.5	1.3 (0.7–2.4)	4.7 (2.5–8.9)	0.8 (0.4–1.6)
No	36	3899.3	0.9 (0.7–1.3)	3.9 (2.8–5.4)	Reference

^a^Other subtypes: Intermediate, posterior, or panuveitis.

Compared to unilateral disease, bilateral uveitis was significantly associated with earlier treatment initiation across all modalities. Specifically, the HR for IDI was 3.1 (95% CI: 1.4–6.8), for systemic prednisolone 5.6 (95% CI: 3.9–8.2), and for DMARDs 11.2 (95% CI: 5.2–23.9). Intermediate, posterior, and panuveitis compared to anterior uveitis, were associated with higher treatment rates, including an HR of 3.5 (95% CI: 2.1–5.8) for periocular corticosteroid injections, 8.0 (95% CI: 3.7–17.4) for IDI, and 8.9 (95% CI: 4.9–16.0) for DMARD initiation (Table 2).

## Discussion

In this study, we examined real-world treatment patterns in NIU over a five-year follow-up period, with particular focus on the timing of therapy initiation. Our findings show that periocular corticosteroid injections and systemic prednisolone were commonly introduced early, with a sharp increase in their use during the first three months of follow-up. In contrast, initiation of intravitreal implants (i.e., IDI) or corticosteroid-sparing agents, despite their demonstrated efficacy, occurred later and less frequently, with gradual uptake over time [5,6]. The later introduction of IDI is notable given its established role as local therapy which provides sustained intraocular corticosteroid delivery for NIU. A recent meta-analysis demonstrated the high efficacy and acceptable safety profile of an intravitreal corticosteroid implant similar to IDI for managing NIU, emphasising their clinical benefit in early disease stages to control chronic inflammation [7,8]. Similarly, a case series by Sisk et al. highlighted favourable outcomes with early IDI intervention, supporting the concept that earlier implementation could improve disease control in patients with NIU and potentially reduce the risk of complications such as UME, epiretinal membrane formation, and elevated intraocular pressure [9]. Our previous work in a Danish NIU cohort demonstrated that most cases of UME developed within the first two years after referral to a tertiary centre, further emphasising the importance of early inflammation control (8.3% developed UME during follow-up; incidence rate 1.4 per 100 person-years [95% CI: 1.3–1.7]; cumulative incidence 10.7% [95% CI: 8.5–13.5]) [10]. Currently, no non-steroidal intravitreal therapies are approved for NIU, leaving IDI and periocular corticosteroid injections as the primary local interventions. Therefore, our findings suggest that barriers for timely use of IDI treatment may exist, including but not limited to logistical challenges, such as the need for an operating room in some centres, and concerns about steroid-related side effects, particularly cataract formation, ocular hypertension, and glaucoma. In our cohort, 191 patients (15.7%) had undergone prior cataract surgery, and 22 patients (1.8%) had undergone prior glaucoma surgery at presentation, with an additional 148 patients (12.2%) and 33 patients (2.7%) undergoing these during follow-up. Given that IDI carries known risks of cataract formation and intraocular pressure elevation, the presence of these risks  may have influenced treatment decisions and contributed to the observed lower uptake. Furthermore, the MUST trial, which compared systemic immunomodulatory therapy to sustained-release intravitreal implant, demonstrated similar uveitis control at 7 years, though the implant group had higher rates of cataract surgery and elevated intraocular pressure [11,12], which may further explain clinician hesitancy regarding the use of IDI in patients with pre-existing ocular comorbidities. The relatively high cumulative incidence of systemic prednisolone (11%) underscores the continued reliance on systemic corticosteroids, often initiated early in the disease course. While studies suggest these can be administered relatively safely, long-term corticosteroid use carries well-known risks [11,12]. In addition, all patients in this study were managed at a centre where biologic therapies such as TNF-α inhibitors were not administered on site for uveitis, requiring referral to specialised centres. Biologic therapies are available and used across Denmark for both uveitis and systemic diseases, however the data were not captured in this dataset, representing a potential confounder which may have influenced the treatment patterns observed in this study. Together, these factors may have contributed to the prolonged reliance on systemic corticosteroids observed in this cohort. DMARD therapy, while less frequently used compared to systemic corticosteroids, showed a steady increase in initiation throughout follow-up even though it typically requires several months to achieve therapeutic effect [13]. A systematic review reported moderate evidence supporting DMARDs for inflammation control in NIU, providing an option to help limit steroid-related complications such as glaucoma and cataract [6]. Current guidelines recommend initiating steroid-sparing therapy when systemic corticosteroids cannot be tapered below 7.5 mg within three months [14]. In our cohort, the median duration of systemic prednisolone treatment was 3.7 months (IQR 1.5–9.7 months), with a mean of 9.1 months, suggesting that some patients may have remained on systemic corticosteroids beyond the recommended duration before transitioning to steroid-sparing therapies. Dosage data were unavailable in our dataset, precluding confirmation of whether the 7.5 mg dosage was met. Taken together, these findings suggest that earlier consideration of DMARDs may be warranted to reduce prolonged corticosteroid exposure. Notably, patients with systemic disease may be more likely to initiate DMARD therapy as part of their disease management, independent of their uveitis. Only DMARDs explicitly documented as initiated for uveitis were included in this study, which may have led to an underestimate of DMARD use in patients where the indication was shared between uveitis and systemic disease. This conservative approach ensures specificity but may partly explain the relatively low DMARD uptake observed in our cohort.

We identified key clinical characteristics associated with more intensive treatment. Bilateral uveitis and non-anterior anatomical subtypes (intermediate, posterior, and panuveitis) were consistently associated with earlier and more frequent treatment initiation across all modalities. These features likely reflect greater inflammatory burden and a higher risk of vision-threatening complications. Consistent with our prior findings in UME, bilateral disease and posterior involvement signal the need for more aggressive therapy, including earlier use of IDI, DMARDs, and biologics [10]. This study use of validated clinical data, with all cases confirmed through detailed medical record review, ensures a high level of diagnostic accuracy and reliability of treatment information [10]. The real-world setting provides a comprehensive view of treatment initiation patterns in NIU, reflecting clinical decision-making in routine practice. The large sample size allowed for subgroup analysis by anatomical subtype and laterality, which yielded clinically meaningful insights. Importantly, our analysis focused on the timing of first treatment initiation rather than treatment frequency or intensity. Some patients may have received multiple therapies in close succession, depending on disease severity and clinician judgement, but our analysis was limited to identifying when each treatment modality was first introduced. Despite its strengths, this study has limitations. First, the included study population reflects individuals with more severe disease manifestations requiring tertiary care, which may limit generalisability to milder NIU cases managed in community settings. Second, the absence of standardised treatment protocols may introduce variability in clinical decision making, as treatment initiation was guided by individual physician judgement rather than uniform criteria. Third, the cohort comprises a predominance of anterior uveitis and unilateral disease, which may inherently result in lower use of corticosteroid-sparing agents, as these therapies are traditionally reserved for cases with systemic disease which is proportionally lower in this cohort. Additionally, we did not assess patient preferences or treatment adherence, which may also influence the timing and selection of therapies. Finally, the analysis did not account for possible contraindications or comorbidities that may have influenced treatment decisions, such as systemic conditions precluding immunosuppression or prior ocular surgeries limiting the use of implants. These factors could partly explain the later introduction of IDI or DMARDs in some cases.

## Conclusion

Our findings highlight a tendency toward early reliance on systemic corticosteroids and periocular corticosteroid injections, with later introduction of IDI and DMARDs. Treatment duration data suggest that corticosteroid therapy may have been continued beyond the durations recommended by current guidelines in a subset of patients. These findings may reflect both the clinical characteristics of this cohort and potential barriers to timely corticosteroid-sparing therapy, highlighting the importance of continued efforts to optimise the timing of treatment in the management of NIU.

## Summary

### What was known before

- Corticosteroids are the first-line treatment for non-infectious uveitis.
- Real world population based data on treatment patterns is limited.

### What this study adds

- Corticosteroids are initially used more frequently while intravitreal dexamethasone implants and DMARDs are introduced more gradually.
- May highlight the need for earlier adoption of corticosteroid-sparing therapies to improve longterm management of non-infectious uveitis.

## Supplementary information

Eye - reporting checklist

## Data Availability

The datasets generated during and/or analysed during the current study are not publicly available due to patient sensitive data.
